# Preliminary Effectiveness of 360° Immersive Virtual Reality for the Acquisition of Empathy-Related Skills in Physiotherapy Students: A Quasi-Experimental Study

**DOI:** 10.1007/s40670-025-02476-8

**Published:** 2026-01-05

**Authors:** María Isabel Gaviña-Barroso, Alberto Bermejo-Franco, Roberto Ucero-Lozano, María Medina-Sampedro, Javier López-Ruiz, Guillermo García-Pérez-de-Sevilla

**Affiliations:** 1 Universidad Europea de Madrid, Faculty of Medicine, Health and Sports, Department of Physiotherapy, Madrid, Spain; 2 Universidad Europea de Madrid, Faculty of Law, Education and Humanities, Aqualab Research Group, Madrid, Spain; 3 Universidad Europea de Madrid, Faculty of Medicine, Health and Sports, Department of Physiotherapy. Therapeutic Exercise and Functional Rehabilitation Research Group, Madrid, Spain; 4 Universidad Europea de Madrid, Faculty of Medicine, Health and Sports, Department of Physiotherapy. Neuroscience and physical therapy Research Group, Madrid, Spain; 5 InHeFis Research Group, Instituto Asturiano de Investigación Sanitaria (ISPA), Oviedo, Spain; 6 Universidad Europea de Madrid, Faculty of Medicine, Health and Sports, Department of Physiotherapy, Women & Health Research Group, Madrid, Spain; 7 Family Phisician. SERMAS. Villaviciosa de Odón Health Center, Villaviciosa de Odón, Madrid, Spain; 8 Universidad Europea de Madrid, Faculty of Medicine, Health and Sports, Department of Real Madrid Graduate School, Madrid, Spain

**Keywords:** Higher education, Physiotherapy, Empathy, Clinical simulation, Virtual reality

## Abstract

**Introduction:**

Virtual reality (VR) simulation programs are emerging as valuable tools in healthcare education, offering immersive experiences that help students develop soft skills like empathy. This study aimed to evaluate the effectiveness of a 360° VR simulation in enhancing empathy among physical therapy students toward patients with acquired brain injury.

**Methods:**

A quasi-experimental study was performed with 3rd year physical therapy students, analyzing their empathy with the Jefferson Scale of Empathy for Health Professionals (JSE-HP) before and after an immersive clinical simulation VR program which allowed the viewer to get first person immersed in a stroke patient’s actual life. A one-way ANOVA of repeated measures was used to determine the PRE-POST intervention differences.

**Results:**

A total of 139 students (58% male; aged 22.31 ± 2.84 years) participated in the VR experience, significantly improving the JSE-HP total score (PRE = 84.65 ± 8.05 versus POST = 86.81 ± 8.11; *p* < 0.01). Comparing the JSE-HP score by sex, significant PRE-POST differences were found in the male participants, but not in the female participants. Both female and male participants improved different items of the cognitive dimension of empathy. In the same line, in the analysis by Spanish level, the native students showed significant improvements in some items relative to the cognitive dimension of empathy.

**Conclusion:**

The VR program could be an educational resource to improve the empathy of health sciences students and thus the future therapeutic relationship with the patient. Male participants and participants with a higher level of Spanish could be more positively affected by this VR program.

**Supplementary Information:**

The online version contains supplementary material available at 10.1007/s40670-025-02476-8.

## Introduction

Current health services emphasize patient-centered care, requiring healthcare professionals to understand patients’ perspectives to improve satisfaction and quality of care [9, 36]. However, modern medicine, technological advancement, and the bureaucratization of health institutions have contributed to a more depersonalized and less human-centered approach to care [29]. To counter this, a shift toward person-centered care is being promoted, emphasizing authenticity, empathy, and respect for patients’ values [38].

Empathy is a key element in building an effective therapeutic relationship. It involves understanding the patient without judgment, recognizing their emotions and concerns, and making them feel heard and accepted [39]. Empathy is a multidimensional concept composed of both cognitive and affective elements that allow individuals to understand and experience others’ emotional states [16, 19, 36]. Importantly, both components of empathy can be developed through education and practice [17].

Empathic relationships in healthcare offer multiple benefits, including greater patient satisfaction, reduced stress, improved treatment adherence, and fewer conflicts between patients and healthcare professionals [3, 36]. Despite its importance, research on empathy in physiotherapy students remains limited, although some studies have addressed this area [11, 32, 43]. In recent years, growing concern about the documented decline in empathy during academic training has led to an increase in educational interventions aimed at strengthening empathy and communication skills in health science students [14, 24, 28, 33]. This decline has been attributed to factors such as emotional exhaustion, academic overload, burnout, and insufficient mentoring [17, 34]. Interventions such as simulation-based learning and role-reversal strategies have demonstrated promising results in fostering empathy, as evidenced by Levett-Jones et al. [24], who reported significant improvements in empathy after students participated in disability perspective simulations [24].

Among the proposed strategies to counteract this decline, clinical simulation has proven to be an effective tool to foster empathy through realistic and emotionally engaging contexts. Specifically, role-reversal simulations—where students adopt the role of the patient—have shown a notable capacity to promote empathy by enhancing perspective-taking and emotional understanding [4]. These experiences allow students to reflect on the patient experience, strengthening their interpersonal competencies and preparing them for more humanistic clinical practice.

Traditionally, simulation in healthcare education has been used to teach technical and psychomotor skills, often through mannequins and anatomical models [2]. However, the integration of immersive technologies such as virtual reality (VR) has opened new opportunities for the development of soft skills, including empathy [45]. VR refers to a fully computer-generated, interactive 3D environment that users can navigate and manipulate in real time, often using headsets and controllers. In contrast, 360° VR involves immersive video captured with omnidirectional cameras, allowing users to explore a scene from a fixed position by looking in all directions, but without the ability to interact with the environment [45].

Traditional educational methods often rely on cognitive perspective-taking, which requires individuals to imagine themselves in another person’s situation. In contrast, VR immerses users directly in realistic scenarios, enabling them to experience situations from another’s perspective in a more engaging and impactful way. Empathy is a multidimensional construct that includes both cognitive empathy—the ability to understand another person’s perspective, thoughts, or mental state—and emotional empathy—the capacity to share or resonate with another person’s emotional experience [36]. This distinction is particularly relevant in the context of VR, as its immersive quality makes it a powerful tool for fostering both dimensions of empathy. Specifically, immersive VR can support both cognitive empathy—by enabling learners to adopt the viewpoint of the patient—and emotional empathy—by triggering affective responses through realistic and emotionally charged scenarios [41].

VR has shown significant potential to enhance empathy toward stigmatized groups, reinforcing its value as an innovative approach to improving interpersonal skills among healthcare professionals. By leveraging VR, healthcare education can move beyond theoretical learning, offering experiential training that strengthens the emotional and social competencies essential for patient-centered care [27].

Thanks to technological advances, clinical simulation has expanded through the incorporation of 360° VR, creating high-fidelity and complex environments that allow students to immerse themselves in realistic clinical contexts safely, without risk to patients [7].

In healthcare, several studies have suggested that immersive virtual simulations may help improve students’ empathy [12, 43]. Although these studies did not specifically employ 360° VR, their results highlight the potential of immersive technologies—such as 360° VR—to foster empathy. Immersive tools like these offer promising opportunities to enhance emotional engagement and understanding in clinical education, particularly when working with complex patient populations such as those with neurological conditions.

For physiotherapy students with limited clinical experience, approaching patients with neurological conditions—such as stroke—can be an overwhelming and emotionally challenging task. Feelings of frustration, uncertainty, and inadequacy may arise when confronting the complexity of these cases, potentially hindering the development of a strong therapeutic relationship, which is essential for effective patient care. To address this, there is a need for educational strategies that not only convey clinical knowledge but also foster empathy by helping students grasp both the cognitive and emotional challenges experienced by patients after a stroke [23]. Despite the increasing integration of immersive technologies in healthcare education, there remains a lack of evidence-based approaches specifically designed to cultivate empathy in physiotherapy students working with this patient population.

The main purpose of this study is to evaluate the effectiveness of “Put Yourself in My Shoes”®, an intervention designed to help physical therapy students personally experience common limitations after stroke, explore their attitudes toward patients’ functionality, quality of life, and social roles, and ultimately develop greater empathy toward this population. Previous research has demonstrated the clinical applicability of VR systems in stroke rehabilitation, aiding professionals in understanding and addressing patients’ functional limitations [23]. Building on this immersive potential, the present study explores VR as a pedagogical tool to foster empathy among future physiotherapists. By focusing on stroke rehabilitation and applying a targeted VR-based intervention, this study contributes to the growing body of literature supporting immersive simulation as a means to promote emotional and cognitive empathy, particularly in health professions where therapeutic relationships are fundamental.

## Methods

### Study Design

A quasi-experimental study was performed with physical therapy (PT) students in a private university in Spain. This study was conducted according to the guidelines of the Declaration of Helsinki and was approved by the Institutional Ethics Committee of the European University of Madrid (study protocol CIPI/213006.61). In addition, this study was reported following both the TREND Reporting Guidelines for Nonrandomized/Quasi-Experimental Study Designs and the key principles of the CONSORT guidelines to ensure transparency and rigor in reporting. The Physiotherapy Degree is offered in two instructional tracks, Spanish and French, which share the same curricular content and, in many cases, the same instructors.

### Setting and Participants

A convenience sample of third-year physiotherapy students was selected for this study. The choice of third-year students was based on the fact that this is when they take the Neurological Physiotherapy course as part of their curriculum. Given that the intervention focused on patients with stroke, we considered it the most appropriate stage for students to grasp key concepts effectively. A total of 139 students (*n* = 139) voluntarily participated in the study, which was conducted during their Neurological Physiotherapy lessons. Participation in the training activity was scheduled as a mandatory component of the course and was non-evaluative. While attendance at the training activity was required, participation in the study itself was entirely voluntary, and students could attend the class without consenting to the study. Nevertheless, all students who attended the training activity chose to participate in the study. Participants were informed about the characteristics, design, and objectives of the study and signed an informed consent form before beginning the protocol. The inclusion criteria for participation were as follows: (a) to be enrolled in Neurological PT course during the academic year 2021/2022, (b) to have had contact with actual patients in the clinical placement course [17], and (c) a minimum level of Spanish B2 according to the Common European Framework of Reference for Languages (CEFR).

### Outcomes and Measurements

An online Google Forms (Menlo Park, CA, USA) survey was used to collect data. Before the start of the simulation session, students completed an anonymous questionnaire to collect data on sex, age, and Spanish language proficiency. These variables were specifically gathered because the degree program is offered in both Spanish and French, while this project was conducted exclusively in Spanish. The aim was to analyze potential differences in comprehension or engagement based on these factors.

Furthermore, before and after the simulation session, students’ empathy was evaluated using the self-applied Jefferson Scale of Empathy for Health Professionals version (JSE-HP) [17] in its validated Spanish version. The scale was translated, culturally adapted, and validated for the Spanish context by Canseco [6]. According to his findings, the JSE-HP showed a Cronbach alpha of 0.82, indicating good internal consistency [17]. Additionally, each item showed a statistically significant positive correlation with the total score, with a median correlation of 0.45. These results support the reliability and construct validity of the instrument in the Spanish population. Cross-validation of the Spanish version has also been confirmed by other authors [1], with findings indicating some cross-cultural differences but overall psychometric robustness.

The JSE-HP comprises 20 items (Annex I). Each item is evaluated using a 7-point Likert scale (1 = strongly disagree, 7 = strongly agree). Ten of these items are written positively and the other ten are negative. Negative items are used to avoid social desirability and acquiescence in the responses. The scale ranges from 20 to 140 points, with higher scores indicating greater empathic orientation [19]. The items 2–4-5–9-10–13-15–16-17 and 20 correspond to the cognitive dimension of empathy, the items 1–7-8–11-12–14-18–19 correspond to the emotional dimension of empathy, and items 3 and 6 correspond to the ability of the individual to put himself in the other’s shoes [17].

The data collected via Google Forms were subsequently exported to Microsoft Excel (Microsoft Corporation, Redmond, WA, USA) for organization and analysis.

### Intervention

All the participants watched an immersive 360° video, a storytelling experience recorded in Spanish, and to enhance the user experience through VR, the video was recorded in high quality and the device was adapted to the visual capability of each participant. The immersive experience was displayed on a Samsung® Galaxy S8 Smartphone (Samsung Electronics, Seoul, South Korea) attached to the VR headset accessory Samsung® Gear VR (Samsung Electronics, Seoul, South Korea). The video, which had a duration of 19 min and 53 s, was based on an immersive VR program called “Put Yourself in My Shoes”*®* (“Ponte en mis zapatos”®), and was designed by PT faculty members with extensive expertise in stroke rehabilitation and patient care. The development of the VR experience involved several phases: an initial planning phase to define the clinical scenarios, followed by the design of the narrative script, preparation of the physical spaces, and a review of key aspects to improve realism and educational impact. The final phase involved video recording conducted in collaboration with a professional production company specialized in immersive content.

Their involvement ensured the clinical accuracy and educational relevance of the simulation, which aimed to evaluate the effect of clinical simulation using VR on physiotherapy students’ empathy. This video allowed the viewer to become immersed in the daily life of a stroke patient from a first-person perspective, including daily activities and medical procedures, displaying the adaptations and limitations that the patient experiences. The goal of this experience was to help students understand how this condition impacts patients’ lives and to foster both cognitive and emotional empathy. A free online trailer version (in Spanish) can be found at the following link: https://youtu.be/bx8ZNuNfmno.

### Statistical Analysis

All the results were analyzed by protocol and intention-to-treat analysis (ITT). The distribution and normality of the data were analyzed with the Kolmogorov–Smirnov test. Data are expressed as mean ± standard deviation. The one-way ANOVA of repeated measures was employed to analyze the PRE-POST intervention difference in the students’ empathy. The level of statistical significance was set at *p* < 0.05. Subgroup analyses were performed to compare results by sex and Spanish proficiency level. These subgroup analyses were interpreted accordingly. Given the multiple comparisons, the Bonferroni correction was applied to adjust significance levels, reducing the likelihood of type I errors. The effect size was assessed with the partial eta squared (*η*^2^), calculated using the sum of squares from the ANOVA model, considering small a value between 0 and 0.05, medium 0.06 to 0.13, and large ≥ 0.14 [35]. All statistical analyses were performed with SPSS 27.0 (IBM, Armonk, NY, USA).

## Results

A total of 139 students (58% male) aged 22.31 ± 2.84 years were recruited, participated in the VR experience, and filled the questionnaires. Concerning the JSE-HP total score, a significant difference was found in the PRE-POST analysis (PRE = 84.65 ± 8.05 versus POST = 86.81 ± 8.11; *p* < 0.01), with a medium effect size (0.06) (Table 1).

**Table 1:** JSE-HP total score subgroup analysis

	PRE	POST	*p*-value	Effect size (***η***^2^)
Total sample (***n*** = 139)	84.65 ± 8.05	86.81 ± 8.11	** < ***0.01*	0.06
Male participants (***n*** = 80)	84.12 ± 8.15	86.84 ± 8.83	*< 0.01*	0.05
Female participants (***n*** = 59)	85.38 ± 7.92	86.78 ± 7.07	*0.07*	0.02
B2 level (***n*** = 42)	85.81 ± 9.33	88.38 ± 10.65	*0.04*	0.05
C1-C2 level (***n*** = 18)	85.28 ± 8.50	88.28 ± 5.81	*0.11*	0.02
Mother tongue (***n*** = 74)	83.66 ± 6.82	85.36 ± 6.77	*0.06*	0.03

Significance was set at *p* < 0.05

Comparing the JSE-HP total score by sex, significant PRE-POST differences were found in the male participants (PRE = 84.12 ± 8.15 versus 86.84 ± 8.83 post; *p* = < 0.01) with a small effect size (0.05), but not in the female participants (PRE = 85.38 ± 7.92 versus POST = 86.78 ± 7.07; *p* = 0.07) (Table 1).

In the JSE-HP total score analysis by Spanish level, there was a significant PRE-POST difference in students with a B2 level (*n* = 42; 30%) (PRE = 85.81 ± 9.33 versus POST = 88.38 ± 10.65; *p* = 0.04), with a small effect size (0.05), but not in the students with a C1-C2 (*n* = 18; 12.9%) (PRE = 85.28 ± 8.50 versus POST = 88.28 ± 5.81 post; *p* = 0.11), nor in the students whose mother tongue was Spanish (*n* = 74; 52.9%) (PRE = 83.66 ± 6.82 versus POST = 85.36 ± 6.77; *p* = 0.06). The data about the Spanish level was missing in five participants, who were not included in this subgroup analysis (Table 1).

Tables 2 and 3 show the PRE-POST JSE-HP score analysis by item, comparing by Spanish level and sex.

**Table 2:** PRE-POST JSE-HP score analysis by item, comparing by Spanish level of the participants

	**B2** (***n*** = 42)		*p*-value	**C1-C2** (***n*** = 18)		*p*-value	Mother tongue (***n*** = 74)		*p*-value
	PRE	POST		PRE	POST		PRE	POST	
**Item 1**	1.95 ± 2.09	2.29 ± 2.42	0.35	1.79 ± 1.69	1.68 ± 1.53	0.68	1.66 ± 1.70	1.40 ± 1.23	0.24
**Item 2**	6.78 ± 0.57	6.76 ± 0.58	0.57	6.68 ± 0.75	6.42 ± 1.43	0.35	6.60 ± 0.72	6.70 ± 0.57	0.23
**Item 3**	3.34 ± 1.59	3.29 ± 1.94	0.80	3.21 ± 1.78	2.21 ± 1.62	0.06	2.84 ± 1.42	2.62 ± 1.54	0.17
**Item 4**	6.51 ± 1.19	6.61 ± 0.92	0.64	6.47 ± 0.77	6.26 ± 1.52	0.49	6.01 ± 1.40	6.56 ± 0.80	0.01
**Item 5**	5.80 ± 1.14	6.17 ± 1.12	0.06	5.47 ± 0.91	5.68 ± 1.53	0.48	5.38 ± 1.52	5.74 ± 1.49	0.01
**Item 6**	2.49 ± 1.43	2.44 ± 1.60	0.86	2.26 ± 1.59	2.53 ± 1.71	0.55	2.21 ± 1.27	2.30 ± 1.42	0.55
**Item 7**	2.24 ± 1.80	2.24 ± 2.03	1.00	1.47 ± 0.70	2.26 ± 1.97	0.14	1.67 ± 1.03	1.59 ± 1.21	0.56
**Item 8**	1.95 ± 1.88	2.17 ± 2.02	0.29	1.89 ± 1.85	1.37 ± 0.96	0.15	1.29 ± 0.84	1.64 ± 1.52	0.03
**Item 9**	5.90 ± 1.36	6.10 ± 1.34	0.36	5.95 ± 1.27	6.53 ± 0.61	0.01	6.36 ± 0.89	6.30 ± 1.19	0.67
**Item 10**	6.34 ± 0.91	6.37 ± 1.02	0.89	6.05 ± 0.97	6.37 ± 1.34	0.38	5.97 ± 1.12	6.44 ± 0.87	0.01
**Item 11**	1.88 ± 1.42	2.12 ± 1.78	0.38	1.79 ± 1.27	1.89 ± 1.52	0.78	1.62 ± 0.91	1.45 ± 0.99	0.06
**Item 12**	2.32 ± 2.13	3.00 ± 2.48	0.16	2.37 ± 1.98	1.95 ± 1.65	0.43	1.82 ± 1.58	2.38 ± 2.32	0.04
**Item 13**	6.27 ± 1.27	6.34 ± 1.30	0.77	6.21 ± 0.98	6.74 ± 0.45	0.01	6.32 ± 0.83	6.60 ± 0.64	0.01
**Item 14**	1.73 ± 1.27	2.07 ± 1.86	0.27	1.47 ± 0.77	1.58 ± 1.26	0.72	1.44 ± 1.04	1.41 ± 1.13	0.85
**Item 15**	5.10 ± 2.08	5.20 ± 2.14	0.80	5.84 ± 1.46	6.16 ± 1.74	0.23	5.93 ± 1.37	6.16 ± 1.24	0.08
**Item 16**	6.39 ± 0.92	6.44 ± 1.16	0.14	6.11 ± 1.05	6.47 ± 0.77	0.17	6.32 ± 1.08	6.70 ± 0.62	0.01
**Item 17**	5.68 ± 1.46	6.07 ± 1.23	0.93	5.79 ± 1.65	6.42 ± 1.07	0.06	5.89 ± 1.16	6.14 ± 1.02	0.07
**Item 18**	4.46 ± 1.54	4.68 ± 1.78	0.49	4.26 ± 1.66	3.95 ± 1.47	0.49	4.64 ± 1.31	4.14 ± 1.44	0.06
**Item 19**	2.44 ± 1.76	2.63 ± 1.65	0.35	2.32 ± 1.34	2.79 ± 1.90	0.14	2.71 ± 1.54	2.52 ± 1.73	0.21
**Item 20**	6.46 ± 0.87	6.54 ± 0.93	0.50	6.37 ± 1.21	6.47 ± 1.26	0.33	6.63 ± 0.89	6.77 ± 0.57	0.21

Significance was set at a *p*<0.05

**Table 3:** PRE-POST JSE-HP score analysis by item, comparing by sex

	Male (***n*** = 80)		*p*-value	Female (***n*** = 59)		*p*-value
	PRE	POST		PRE	POST	
**Item 1**	2.09 ± 2.06	1.95 ± 1.98	0.56	1.41 ± 1.48	1.50 ± 1.57	0.69
**Item 2**	6.61 ± 0.73	6.56 ± 0.89	0.59	6.79 ± 0.55	6.88 ± 0.38	0.09
**Item 3**	3.05 ± 1.51	2.83 ± 1.73	0.18	3.22 ± 1.64	2.84 ± 1.76	0.09
**Item 4**	6.09 ± 1.28	6.39 ± 1.05	0.04	6.53 ± 1.19	6.76 ± 0.73	0.21
**Item 5**	5.40 ± 1.42	5.73 ± 1.51	0.02	5.78 ± 1.17	6.03 ± 1.23	0.02
**Item 6**	2.32 ± 1.40	2.51 ± 1.61	0.34	2.38 ± 1.52	2.21 ± 1.27	0.33
**Item 7**	1.88 ± 1.33	2.05 ± 1.73	0.31	1.74 ± 1.41	1.62 ± 1.40	0.62
**Item 8**	1.56 ± 1.28	1.78 ± 1.48	0.14	1.69 ± 1.74	1.69 ± 1.80	1.00
**Item 9**	6.01 ± 1.24	6.21 ± 1.22	0.18	6.16 ± 1.28	6.33 ± 1.10	0.29
**Item 10**	6.05 ± 1.10	6.21 ± 1.07	0.20	6.21 ± 0.93	6.62 ± 0.86	0.01
**Item 11**	1.90 ± 1.23	1.95 ± 1.46	0.73	1.64 ± 1.28	1.34 ± 1.12	0.13
**Item 12**	2.02 ± 1.70	2.50 ± 2.21	0.07	2.05 ± 2.00	2.34 ± 2.36	0.42
**Item 13**	6.12 ± 1.13	6.38 ± 1.12	0.11	6.59 ± 0.65	6.66 ± 0.79	0.47
**Item 14**	1.74 ± 1.28	1.82 ± 1.48	0.67	1.28 ± 0.62	1.43 ± 1.34	0.38
**Item 15**	5.54 ± 1.53	5.73 ± 1.33	0.04	5.66 ± 1.95	5.76 ± 1.93	0.69
**Item 16**	6.12 ± 1.14	6.49 ± 0.77	< 0.01	6.57 ± 0.79	6.72 ± 0.91	0.06
**Item 17**	5.78 ± 1.31	6.04 ± 1.19	0.07	5.90 ± 1.32	6.34 ± 0.89	0.02
**Item 18**	4.57 ± 1.44	4.15 ± 1.48	0.06	4.67 ± 1.51	4.41 ± 1.69	0.22
**Item 19**	2.78 ± 1.65	2.80 ± 1.79	0.85	2.43 ± 1.52	2.41 ± 1.59	0.93
**Item 20**	6.44 ± 0.94	6.54 ± 0.93	0.24	6.69 ± 0.92	6.86 ± 0.51	0.16

Significance was set at *p* < 0.05

Concerning the PRE-POST JSE-HP score analysis by item comparing by Spanish level of the participants, the mother tongue group showed significant improvements in the items 4, 5, 8, 10, 12, 13, and 16 (*p* < 0.05). The C1-C2 group improved the items 9 and 13 score (*p* < 0.05), while the B2 group did not show any significant change in any of the individual items (*p* > 0.05).

Regarding the PRE-POST JSE-HP score analysis by item comparing by sex, the male participants showed significant improvements in the items 4, 5, 15, and 16 (*p* < 0.05), while the female participants improved in the items 5, 10, and 17 post-intervention (*p* < 0.05).

## Discussion

The objective of this study was to analyze the effectiveness of an experiential learning program based on simulation with 360° VR called “Put Yourself in My Shoes”® to increase empathy among physical therapy students toward patients with brain damage. Additionally, this study examined whether differences in empathy outcomes were associated with sex or the students’ Spanish proficiency level.

The results demonstrated statistically significant differences between pretest and posttest empathy scores, suggesting that the VR simulation could increase empathy in physiotherapy students. This finding aligns with previous studies by Ward et al. [43] and Everson et al. [12], which reported improvements in empathy among physiotherapy and nursing students using VR-based role-playing. However, those studies employed different measurement tools than the JSE-HP. Given the importance of measurement instruments in VR-based empathy training, future studies could explore the use of alternative measures such as the Interpersonal Reactivity Index (IRI), which assesses multiple dimensions of empathy and has been employed in VR-related research [8]. Notably, the JSE-HP is one of the few instruments specifically designed for healthcare contexts. Unlike more general empathy scales, it assesses both cognitive components (e.g., perspective-taking) and emotional responsiveness in clinical interactions, making it particularly suitable for evaluating educational interventions in healthcare [12, 43].

Another notable finding was the low overall empathy scores observed in this study, both at baseline (84.65 ± 8.05) and after the VR intervention (86.81 ± 8.11), which fall below the reference values, considering a low empathy level scores below 95 in men and below 100 in women [18]. This contrasts with other studies using the JSE-HP, which have reported higher empathy scores in physiotherapy [11, 30, 32] and other health science disciplines [5, 10, 18]. Several methodological factors may account for this discrepancy, including the absence of randomization and differences in students’ Spanish proficiency, which may have influenced their immersion in the simulation. These elements potentially affected engagement and comprehension during the intervention, thereby impacting empathy scores. Although students’ prior independent experience with VR is unknown, the curriculum confirms that VR technology had not been previously used in their training, which suggests that the VR intervention was likely a novel experience for most participants. These factors could have impacted how engaged students were with the VR experience and, consequently, their empathy scores.

Regarding sex differences, previous literature has often found that women exhibit higher empathy levels than men [18, 25, 30]. However, findings on this topic remain mixed, particularly among health sciences students. In our study, male participants showed a statistically significant increase in empathy scores after the VR intervention, while no significant change was observed among female participants. Although this pattern differs from what has been reported in some previous interventions [10, 21, 32], it is important to interpret these results cautiously, as they may be influenced by factors such as baseline empathy levels, sample size, or varying engagement with the simulation.

While our study did not directly assess emotional self-awareness, socialization patterns, or neurobiological variables, we briefly reference these factors as potential interpretations drawn from existing literature. These interpretations are presented cautiously and for context only, without claiming causal inference. For example, a study on adolescents using the IRI and the Toronto Alexithymia Scale found that girls scored higher on emotional concern, personal distress, and fantasy. However, girls also reported greater difficulty identifying feelings and less externally oriented thinking than boys. This difficulty in identifying emotions may impair their ability to differentiate between their own emotions and those of others, potentially leading to more self-focused and aversive responses when confronted with others’ suffering. Conversely, boys’ externally oriented thinking may help mitigate personal distress when faced with others’ discomfort [37, 42].

In the context of immersive experiences, such as VR, these differences in emotional processing may lead to distinct patterns of engagement. Some evidence suggests that immersive technologies may be especially effective among individuals with lower baseline emotional engagement or empathy scores, often observed in male students [22]. This might partially account for the greater empathy gains observed in male participants in our study, although this interpretation should be approached with caution and warrants further investigation.

Although speculative, these insights serve to frame our findings within broader theoretical perspectives. The observed differences might be linked to socialization processes and gender role expectations [20], or neurobiological factors such as differential activation of mirror neurons and interhemispheric connectivity [31]. Empathy is a multi-layered phenomenon with varying degrees of complexity that unfolds throughout development. Different components of empathy (i.e., affective, cognitive, and prosocial motivation) interact and are expressed behaviorally. Studies indicate that significant sex differences in empathy arise from both bottom-up (e.g., neurobiological structures, genetic, and hormonal factors) and top-down processes (e.g., socialization, environmental influences). Nevertheless, we recognize that these frameworks exceed the scope of our data and are offered here to guide future research rather than to explain our specific results. While our findings indicate a greater empathy improvement in males compared to females, it is crucial to contextualize this result within existing theoretical frameworks. One possible explanation is that males, starting from a baseline empathy score that appears lower than expected, may show greater room for improvement when exposed to empathy-enhancing interventions.

Additionally, socialization differences may play a role; while females are often encouraged to express empathy from an early age, males may receive less reinforcement for empathetic behaviors. Variations in self-reported empathy measures, cultural factors, and academic stressors might also contribute to the observed baseline levels, necessitating a nuanced interpretation of these findings. Although these hypotheses are tentative and not derived from our dataset, they suggest that interventions promoting perspective-taking and emotional attunement—particularly through experiential or immersive formats—might have a more pronounced effect on males, who may not have previously received strong reinforcement for developing such skills [15, 44].

Notably, the items with significant changes pertained to the cognitive dimension of empathy for both sexes (items 5,10,16,17), while male students also showed significant improvements in the emotional dimension (item 18). These results suggest that “Put Yourself in My Shoes”® may be particularly effective in eliciting empathic changes in male students, possibly reflecting different ways in which VR experiences engage cognitive and emotional processes across genders. Future studies should explicitly investigate these possible moderating variables to better understand the mechanisms underlying sex/gender differences in empathy development [37, 42].

When analyzing empathy levels by Spanish proficiency, students with native-level Spanish exhibited significant improvements in items related to both cognitive (items 4–5-10–13-15–16-17–20) and emotional empathy (items 8 and 18), as well as perspective-taking (item 3). However, the distribution of students by Spanish level was not homogeneous, limiting the generalizability of these findings. Interestingly, this finding is somewhat counterintuitive, as one might expect non-native speakers to benefit more from immersive experiences as a way to bridge linguistic and cultural gaps. Given the role of linguistic proficiency in immersive learning experiences, this suggests that a higher command of the language may allow for more nuanced comprehension of emotional and contextual cues within the simulation. Native speakers may have been better equipped to decode tone, idiomatic expressions, or cultural references embedded in the scenarios, facilitating deeper empathetic engagement.

Conversely, non-native speakers may have faced linguistic barriers that reduced their ability to fully engage with the emotional content of the simulation. This raises questions about whether simulations offered only in one language may inadvertently disadvantage certain learners. Future studies could investigate whether offering simulations in multiple languages enhances empathy outcomes among non-native speakers, and whether adaptations in linguistic complexity or the addition of subtitles might facilitate more equitable immersion.

## Limitations

The present study has several limitations. Firstly, there was no randomization of participants, as all eligible students who consented were included, which may have introduced selection bias and limits the generalizability of the results. Secondly, the study did not include a control group; as this was an exploratory phase, the focus was on offering the intervention to all participants. However, the absence of a control group makes it difficult to isolate the effects of the VR experience from other potential influences on empathy. In particular, without comparing the VR experience to conventional formats (e.g., video, reading materials, or lectures), we cannot attribute the observed gains specifically to the immersive nature of the intervention.

Additionally, our study assessed empathy levels only in the short term, without evaluating whether the observed improvements were maintained over time. Understanding the long-term impact of such interventions is crucial to determine their true educational value and lasting influence on professional development. Future studies should incorporate longitudinal assessments to evaluate the durability of the intervention’s effects. Moreover, while we measured self-reported empathy changes, we did not assess whether these translated into actual behavioral changes in clinical practice. Future research should include observational or patient-reported measures to determine whether increased empathy scores correspond to tangible improvements in patient interactions.

Another limitation of this study is the variability in participants’ Spanish proficiency (B2, C1-C2, and native speakers), which may have influenced their immersion in the simulation, their understanding of the intervention, and their responses to the Spanish-language questionnaires. Although we conducted a stratified analysis to address this factor, future studies could explore strategies to minimize its impact, such as adapting linguistic materials or assessing comprehension levels. Additionally, the study focused on a single neurological condition (brain damage), limiting the generalizability of findings to other clinical scenarios. Expanding the research to include different neurological conditions could provide a broader understanding of VR-based empathy training effectiveness.

Moreover, the present study lacks comparison with similar VR-based empathy training interventions for physiotherapy students. Most existing studies either use different empathy scales [12, 43] or assess empathy levels without implementing targeted interventions [11, 13, 32]. Expanding the research scope to include different neurological conditions and assessing additional variables such as students’ motivation, prior knowledge of brain damage, and affinity for technology could provide deeper insights into the effectiveness of VR-based empathy training [26].

### Clinical Implications and Future Research Lines

Despite these limitations, this study holds relevance for both educational practice and research. Future research should consider assessing empathy after the debriefing rather than immediately following the simulation, as post-simulation reflections may enhance empathy development. Additionally, it would be valuable to explore the relationship between empathy and compassion, given that compassion reflects the transition from empathetic understanding to prosocial action. Long-term retention of empathy gains also warrants investigation to determine whether VR simulations foster durable attitudinal changes. Moreover, assessing variables such as satisfaction and usability could offer critical insights for refining and adapting the intervention to diverse learning contexts.

As VR continues to evolve as an educational tool, future studies should aim to improve methodological rigor by employing randomized controlled trial designs. Incorporating control groups and randomization would help isolate the specific effects of immersive experiences from other influencing variables, providing stronger causal inferences and more robust evidence to guide curricular development. Longitudinal follow-up is also recommended to evaluate whether observed improvements in empathy are sustained over time and translate into lasting changes in clinical behavior.

Another important consideration for future research involves the choice of measurement instruments. While this study employed the JSE-HP, future studies could explore the use of complementary tools such as the IRI, which captures a wider range of empathy dimensions and has been used in VR-related empathy research [28]. Comparing general and healthcare-specific empathy scales could yield valuable information about the sensitivity and appropriateness of each tool in immersive educational contexts.

Lastly, further investigations should explore how individual factors—such as students’ motivation, familiarity with neurological conditions, language proficiency, and affinity for technology—may influence their engagement with VR simulations and their empathy-related outcomes [26, 40]. By accounting for these variables, future interventions can be better tailored to maximize educational impact and foster meaningful empathic development in health professions education.

## Conclusions

The results of this study suggest that the VR program “Put Yourself in My Shoes”® may serve as a useful educational tool to enhance empathy among health sciences students, particularly physiotherapy students, potentially contributing to improved future therapeutic relationships with patients. While some subgroup differences—such as greater benefit among male participants and those with higher Spanish proficiency—were observed, these findings should be interpreted cautiously due to the study’s design limitations, including the absence of a control group. Furthermore, although significant PRE-POST improvements were observed in males but not females, baseline empathy scores indicated males started from a slightly lower point, suggesting that males may have had more room for improvement. This baseline difference has been considered in the interpretation but should be further explored in future studies to better understand sex-related responses to VR interventions. Given the specific needs of physiotherapy students, further research is needed to confirm these results and to explore the program’s impact across diverse student populations. Future studies should also examine additional factors such as prior clinical experience and cultural background to better understand and optimize the effectiveness of VR-based empathy training in health sciences education.

## Supplementary Information

Below is the link to the electronic supplementary material.

ESM 1 (XLSX 37.1 KB)

## Data Availability

For full video link, contact corresponding author. The raw data have been attached as supplementary material. Should you need any further information, contact corresponding author: alberto.bermejo@universidadeuropea.es.

## Declarations

### Disclaimer

The lead author María Isabel Gaviña Barroso affirms that this manuscript is an honest, accurate, and transparent account of the study being reported; that no important aspects of the study have been omitted; and that any discrepancies from the study as planned (and, if relevant, registered) have been explained.
