# Author Correction: Preliminary Effectiveness of 360° Immersive Virtual Reality for the Acquisition of Empathy-Related Skills in Physiotherapy Students: A Quasi-Experimental Study

**DOI:** 10.1007/s40670-026-02681-z

**Published:** 2026-02-24

**Authors:** María Isabel Gaviña-Barroso, Alberto Bermejo-Franco, Roberto Ucero-Lozano, María Medina-Sampedro, Javier López-Ruiz, Guillermo García-Pérez-de-Sevilla

**Affiliations:** 1 Faculty of Medicine, Health and Sports, Department of Physiotherapy, Universidad Europea de Madrid, Madrid, Spain; 2 Faculty of Law, Education and Humanities, Aqualab Research Group, Universidad Europea de Madrid, Madrid, Spain; 3 Faculty of Medicine, Health and Sports, Department of Physiotherapy. Therapeutic Exercise and Functional Rehabilitation Research Group, Universidad Europea de Madrid, Madrid, Spain; 4 Faculty of Medicine, Health and Sports, Department of Physiotherapy. Neuroscience and physical therapy Research Group, Universidad Europea de Madrid, Madrid, Spain; 5 InHeFis Research Group, Instituto Asturiano de Investigación Sanitaria (ISPA), Oviedo, Spain; 6 Faculty of Medicine, Health and Sports, Department of Physiotherapy, Women & Health Research Group, Universidad Europea de Madrid, Madrid, Spain; 7 Family Phisician. SERMAS. Villaviciosa de Odón Health Center, Villaviciosa de Odón, Madrid, Spain; 8 Faculty of Medicine, Health and Sports, Department of Real Madrid Graduate School, Universidad Europea de Madrid, Madrid, Spain

**Author Correction: Medical Science Educator**

**https://doi.org/10.1007/s40670-025-02476-8**

The authors failed to secure permission from the copyright holder before using the JSE in their project. After contacting the copyright holder, scoring methods and use of the scale were verified and used without modification. Retrospective permission has been received from Thomas Jefferson University. Consent for this correction has been obtained from all authors.

